# Robotic Versus Laparoscopic Colorectal Resection: Are We There Yet?

**DOI:** 10.7759/cureus.19698

**Published:** 2021-11-18

**Authors:** Salah Abdel Jalil, Ala’ Abdel Abdel Jalil, Rachel Groening, Saptarshi Biswas

**Affiliations:** 1 General Surgery, Grand Strand Medical Center, Myrtle Beach, USA; 2 Gastroenterology and Hepatology, Cleveland Clinic Foundation, Cleveland, USA; 3 Trauma and Acute Care Surgery, Grand Strand Regional Medical Center, Myrtle Beach, USA

**Keywords:** diverticulitis, rectal surgery, laparoscopic, robotic, colon surgery

## Abstract

Background: Laparoscopy-assisted surgery (LAS) for colorectal cancer (CRC) was first described in 1991 and robotic-assisted surgery (RAS) for CRC was first reported in 2002; robotic-assisted colorectal surgery (RACS) is becoming increasingly popular. However, data comparing its outcomes to other established techniques remain limited to small case series. Our primary goal was to review the mortality outcome difference between laparoscopic versus robotic elective colon resection at a small, community hospital.

Study design: We conducted a retrospective review of 2089 patients at the South Atlantic division for cases who underwent robotic and laparoscopic colectomies at our division in 2014-2018. All cases were elective surgeries and analysis was performed within these two subgroups.

Results: In this study, 306 patients underwent robotic colorectal surgery versus 1783 patients who underwent laparoscopic-assisted colorectal surgery. Readmission rate within 30 days of operation was significantly lower for laparoscopic-assisted colorectal resection (LACR) versus RACS (445.4% vs. 53.9%, p= 0.006). However, the length of hospital stay was significantly shorter for RACS with a median of three days (interquartile range {IQR}: 2-5) versus four days (IQR: 3-7) for LACR (p=0.0001). There were no significant differences between the two groups for post-operative incisional hernias, anastomotic leaks, post-operative pain control, surgical site infections, or rate of conversion to an open procedure.

Conclusion: Our study showed a similar outcome between LACR and RACS for post-operative incisional hernias, anastomotic leaks, post-operative pain control, surgical site infections, and rate of conversion to an open procedure. Also, our study showed a readmission rate within 30 days of operation was significantly lower for LACR versus RACS. However, the length of hospital stay was significantly shorter for RACS with a median of three days when compared to LACR. Future research should focus on surgeon-specific variables, such as comfort, ergonomics, distractibility, and ease of use, as other ways to potentially distinguish robotic from laparoscopic colorectal surgery.

## Introduction

The surgical resection of colorectal disease processes has been significantly advanced over the last three decades. With the advent of laparoscopic surgery in 1991, the surgical intervention of colorectal disease has become less invasive leading to decreased hospital stays and shorter recovery times, compared to conventional, open surgery [1]. With the advent of robotic surgery in 2002, these minimally invasive techniques were superior to old open techniques. Even though laparoscopic surgery has improved the surgical management of colorectal disease, robotic surgery has quickly surpassed laparoscopic surgery by improving the surgeon’s ability to perform complex colorectal resections [2]. The many hypothesized advantages of robotic-assisted colorectal surgery (RACS), different from that of laparoscopic surgery, include a more stable three-dimensional view, an increased dexterity for maneuvering instruments with better ergonomics, and physiologic tremor filtering [3]. Robotic-assisted colorectal surgery (RACS) is becoming increasingly popular, but data comparing outcomes to other established techniques remain limited to small case series [4]. Interest in expanding the robot's application to colorectal procedures has been driven in part by the theoretical technological advancements it has over conventional laparoscopy. In addition to improving the technical ability of the surgeon to perform complex colon resections, the literature supports clinical benefits for the patients [5]. Studies have shown that robotic surgery results in shorter hospital stays, lower rate of conversion to open surgery, and lower rates of intraoperative complications [6]. There is still a great deal that we need to know before we fully understand the benefits of robotic surgery for surgical management of colorectal disease, among which are lower conversion rates to open surgery, better oncological outcomes, and nerve preservation [5].

This study aimed to compare the efficacy and safety of elective robotic-assisted colon resection (RACS) versus standard laparoscopic-assisted colorectal resection (LACR) in patients with either diverticulitis or colorectal cancer. Secondary outcomes included length of hospital stay, surgical site infection, incisional hernia, post-operative anastomotic leak, post-operative pain control, and rate of conversion to open procedure.

This article was previously presented as an abstract at the 2021 Southeastern Surgical Congress (SESC) on August 24, 2021.

## Materials and methods

We performed a retrospective review of the Health Care Administration (HCA) enterprise data warehouse was conducted for patients at a regional community hospital between 2014 and 2018 at the South Atlantic division. Adult patients were between the ages of 50 and 80 years who had received elective colorectal resection (laparoscopic, “LACR” or robotic, “RACR”) for either complicated diverticulitis or colorectal cancer, as identified by the International Classification of Diseases (ICD)-10 codes. All open colorectal surgery cases were excluded. The occurrence of incisional hernia, post-operative anastomotic leak, and mortality was obtained to evaluate the primary outcome of safety. Secondary outcomes included length of hospital stay, readmission rate within 30 days of surgery, surgical site infection, conversion to open procedure, and post-operative pain control (i.e., administration and number of pain medications). Comparisons of categorical variables between surgery types were performed using chi-square tests, while differences in numerical variables were evaluated using Mann-Whitney U tests. Bivariate and omnibus tests relied on α=0.05 to determine statistical significance, and necessary post hoc comparison tests used a Bonferroni adjustment for α. Statistical analyses were performed using Statistica software (Palo Alto, CA: TIBCO Software Inc.).

## Results

Readmission rate within 30 days of operation eight was significantly lower for LACR versus RACR (45.4% vs. 53.9%, p=0.006). The length of hospital stay was significantly shorter for RACR with a median of three days (interquartile range {IQR}: 2-5) versus four days (IQR: 3-7) for LACR (p= 0.0001). There were no significant differences between LACR and RACR for post-operative incisional hernias, anastomotic leaks, post-operative pain control, surgical site infections, or rate of conversion to an open procedure.

Between 2014 and 2017, a total of 2089 patients with diverticulitis or colorectal cancer underwent elective colorectal resections. Among these, 1738 (85.35%) were LACR and 306 (14.65%) were RACR. Significantly, more robotic resections occurred in later (2016-2017) than in earlier years (2014-2015; χ^2^=67.86, df=4, p<0.0001), but there were statically significant differences in surgery type by sex (χ^2^=3.75, df=1, p=0.05) as more female underwent RACR surgery, while more male had LACR surgery, but there was no difference in the race (χ^2^=2.40, df=3, p=0.49; Table 1). There was no significant difference in median age between patients in RACR (66 years) and LACR (67 years; z=-0.87, p=0.39). A high proportion of resections were inpatient encounters (RACR=95.10%, LACR=99.16%; Table 1). Overall, few patients experienced any of the primary outcomes, including incisional hernia (0.19%), post-anastomotic leak (1.10%), or mortality (0.43%; Table 1).

**Table 1:** Patient characteristics of laparoscopic and robotic groups RACR: robotic-assisted colorectal resection; LACR: laparoscopic-assisted colorectal resection

Variable	Overall, n (%)	RACR, n (%)	LACR, n (%)	p-Value
Sex (n=2086)				
Female	1166 (55.90)	186 (60.98)	980 (55.03)	0.05
Male	920 (44.10)	119 (39.02)	801 (44.97)	
Race (n=2084)				
Black	180 (8.64)	30 (9.84)	150 (8.43)	0.49
White	1807 (86.71)	265 (86.89)	1542 (86.68)	
Other	93 (4.46)	10 (3.28)	83 (4.67)	
Hispanic	4 (0.19)	0 (0.00)	4 (0.22)	
Year				
2014	551 (26.38)	44 (14.38%)	507 (28.44%)	<0.0001
2015	518 (24.80)	52 (16.99%)	466 (26.14%)	
2016	359 (17.19)	58 (18.95%)	301 (16.88%)	
2017	328 (15.70)	69 (22.55%)	259 (14.53%)	
2018	333 (15.94)	83 (27.12%)	250 (14.02%)	

Furthermore, the occurrence of these events did not differ by surgery type (incisional hernia: 0.17%, p=0.56; post-anastomotic leak: 1.07%, p=0.71; mortality: 0.37%, p=0.76; Tables 2, 3). Regarding secondary outcomes, the occurrence of surgical site infection and readmission mortality were low among all patients, 0.77% and 0.31%, respectively. There were no significant differences in the occurrence of surgical site infection (0.65% vs. 0.79%; χ^2^=0.06, df=1, p=0.81) or readmission mortality (0.00% vs. 0.37%; χ^2^=0.60, df=1, p=0.44) between RACR and LACR patients (Tables 2, 3). Overall, 80.61% of patients received post-op pain medication, and there was no significant difference between surgical groups (RACR=78.43%, LACR=80.99%; χ^2^=1.09, df=1, p=0.30). Similarly, the median number of pain medications received did not significantly differ between surgery groups (both groups=6; z=0.39, p=0.69; Tables 2, 3). Readmission rate within 30 days of operation was significantly lower for LACR than RACR (45.43% vs. 53.92%; χ^2^=7.57, df=1, p= 0.006), while the length of hospital stay was significantly shorter for RACR with a median of three days (IQR: 2-5) versus four days (IQR: 3-7) for LACR (z=-6.71, p<0.0001; Tables 2, 3).

**Table 2:** Patient outcomes of laparoscopic and robotic groups RACR: robotic-assisted colorectal resection; LACR: laparoscopic-assisted colorectal resection

Variable	Overall, n (%)	RACR, n (%)	LACR, n (%)	p-Value
Primary				
Expired	9 (0.43%)	1 (0.33%)	8 (0.45%)	0.76
Incisional hernia	4 (0.19%)	1 (0.33%)	3 (0.17%)	0.56
Post anastomotic leak	23 (1.10%)	4 (1.31%)	19 (1.07%)	0.71
Secondary				
Surgical site infection	16 (0.77%)	2 (0.65%)	14 (0.79%)	0.81
Post-operative pain medication	1684 (80.61%)	240 (78.43%)	1444 (80.99%)	0.30
Readmission within 30	975 (46.67%)	165 (53.92%)	810 (45.43%)	0.006
Readmission mortality	3 (0.31%)	0 (0.00%)	3 (0.37%)	0.44

**Table 3:** The median of patient characteristics and outcomes of laparoscopic and robotic groups RACR: robotic-assisted colorectal resection; LACR: laparoscopic-assisted colorectal resection; IQR: interquartile range

Variables	Median (IQR)	p-Value
Age		
Overall (n=2,089)	67.00 (60.00-72.00)	0.39
RACR (n=306)	66.00 (60.00-71.00)	
LACR (n=1,783)	67.00 (60.00-72.00)	
Length of stay		
Overall (n=2,089)	4.00 (3.00-6.00)	<0.0001
RACR (n=306)	3.00 (2.00-5.00)	
LACR (n=1,783)	4.00 (3.00-7.00)	
Post-operative pain medicines		
Overall (n=1,684)	9.10 (10.75); 6.00 (3.00-12.00); 1.00-151.00	0.69
RACR (n=240)	9.35 (10.53); 6.00 (3.00-12.00); 1.00-71.00	
LACR (n=1,444)	9.06 (10.79); 6.00 (3.00-11.00); 1.00-151.00	

## Discussion

Minimally invasive surgery has changed the course of surgical intervention for colorectal disease, allowing surgeons to perform complex colorectal resections with ease. Of course, there is a learning curve in mastering the robot, but studies have shown surgeons can easily acquire the skill [6]. As minimally invasive surgery progresses, new technology is separating laparoscopic from robotic surgery. The introduction of robotic technology into modern-day surgery has the potential to offer significant advantages. The system translates the natural movements of the surgeon's hands into precise laparoscopic instrument movements inside the patient [7]. But despite the advancements in this technology, there are still disadvantages such as higher costs and length of the procedure [8,9]. Studies have shown that robotic surgery tends to take longer than laparoscopically due to the assembly and use of the robot [3]. Others have also shown increased complications in frail populations [6]. As innovations are developed, these disadvantages will hopefully be resolved. Until then we will continue to assess how these techniques are advancing minimally invasive surgery.

Multiple studies have been conducted showing the differences between laparoscopic and robotic surgeries, but they have been of small sample sizes [10]. Their differences highlight the advancement of robotic surgery in the surgical intervention of colorectal disease. In the present study, we had the opportunity to evaluate colorectal procedures and the role of robotic and laparoscopic surgeries. We specifically evaluated readmission rates within the last 30 days, post-operative complications, and length of hospital stay.

Our analysis took place between 2014 and 2017, where 2089 patients who underwent colorectal surgery for either diverticulitis or colorectal cancer were evaluated. The group was then divided into 1738 surgeries performed laparoscopically and 306 performed robotically. We understand the potential limitation of the different sample sizes between the two groups and the diversity of cases performed. In addition, our populations were restricted to diverticular disease and colorectal cancer. This difference between sample sizes in the two groups could be due to the fact that more surgeons are trained in laparoscopic surgery instead of robotic surgery. From our data review, we found that patients who underwent LACR had significantly lower 30-day readmission rates compared to the RACR group. In contrast, a meta-analysis by Trinh et al. showed that there was no statistical significance in admit rate between these two groups [3]. We realize this difference could be attributed to our modest sample size. Even though LACR had lower readmission rates compared to RACR, the robotic patients had shorter hospital stays with a median of three days versus four days in comparison to LACR. This is consistent with other meta-analyses investigated on this topic, showing that there was a decreased length of stay with robotic surgery compared to laparoscopic [2].

In this analysis, we also reviewed the primary complications such as incisional hernias, anastomotic leak, post-operative pain, surgical site infections, and conversion to open comparing RACR and LACR. The incisional hernia complications were reviewed over a three- to six-year time span from the initial surgery. We found that there was no significant difference between the two groups in regards to these primary complications. This finding was somewhat different from what has been reported previously. For example, Trinh et al. reported an increased conversion to open with robotic surgery versus laparoscopic surgery [3]. These same authors found no statistical difference in overall primary complications between RACR and LACR, which was consistent with our findings [3].

We did not investigate the length of the operative time when comparing robotic and laparoscopic surgeries. However, other studies have shown that robotic surgery results in a longer operative time when compared to laparoscopic surgery [9]. In our study, we found that a readmission rate within 30 days of operation was significantly lower for LACR when compared to RACS. However, the length of hospital stay was significantly shorter for RACS when compared to LACR with a median of three days; when reviewing the literature, we appreciated similar yet some different results. But it was also noted that many of the meta-analyses conducted included more laparoscopic cases versus robotic cases for surgical intervention of colorectal disease. According to Halabi et al., intestinal anastomosis methods using totally robotic surgery result in a shorter time to bowel function recovery and tolerance to a solid diet [4]. Also, the study by Liao et al. showed that conversion rates and times to the recovery of bowel function were significantly reduced following RACS compared with LACR. And there were no significant differences in complication rates, lengths of hospital stays, proximal margins, distal margins, or harvested lymph nodes between the two techniques [2]. Because of this finding, we feel that there is still much room for a better understanding of how beneficial the use of robotic surgery is for the surgical management of the colorectal disease. With the results already obtained, we can clearly appreciate that minimally invasive surgery is beneficial to the surgeon and the patient in multiple ways.

## Conclusions

Robotic-assisted colorectal resection (RACR) was not inferior to laparoscopic-assisted colorectal resection (LACR) in patients with either diverticulitis or colorectal cancer. LACR was significantly associated with a lower 30-day readmission rate, but RACR was associated with a slightly shorter hospital stay. Our results reflect those in other studies and reveal that more research needs to be conducted comparing these two minimally invasive techniques. As more surgeons become trained on the robot, the more we will be able to explore this topic. Further research utilizing prospective methods, with a focus on surgeon-specific variables such as comfort, ergonomics, distractibility, and ease of use, is of paramount need to elucidate further comparative outcomes of RACR versus LACR.
